# Reduced Immunogenicity of Intraparenchymal Delivery of Adeno-Associated Virus Serotype 2 Vectors: Brief Overview

**DOI:** 10.2174/1566523221666210922155413

**Published:** 2022-03-09

**Authors:** Wuh-Liang Hwu, Shin-ichi Muramatsu, Bruria Gidoni-Ben-Zeev

**Affiliations:** 1 Department of Medical Genetics and Pediatrics, 7 Chung-Shan S. Road, National Taiwan University Hospital, Taipei, Taiwan;; 2 Division of Neurological Gene Therapy, Center for Open Innovation, Jichi Medical University, 3311-1 Yakushiji, Shimotsuke, Tochigi 329-0498, Japan;; 3 Center for Gene & Cell Therapy, The Institute of Medical Science, The University of Tokyo, 4-6-1 Shirokanedai, Minato, Tokyo 108-0071, Japan;; 4 Department of Pediatric Neurology, Sackler School of Medicine, Tel Aviv University, Tel Aviv-Yafo, 6997801, Israel

**Keywords:** Adeno-associated virus, aromatic l-amino acid decarboxylase deficiency, eladocagene exuparvovec, immunogenicity, vectors, rare disease

## Abstract

Pre existing immunity to adeno-associated virus (AAV) poses a concern in AAV vector–mediated gene therapy. Localized administration of low doses of carefully chosen AAV serotypes can mitigate the risk of an immune response. This article will illustrate the low risk of immune response to AAV serotype 2 vector–mediated gene therapy to the brain with support from clinical trial data in aromatic L-amino acid decarboxylase deficiency and Parkinson disease.

## 1 INTRODUCTION

**Fig. (1):**
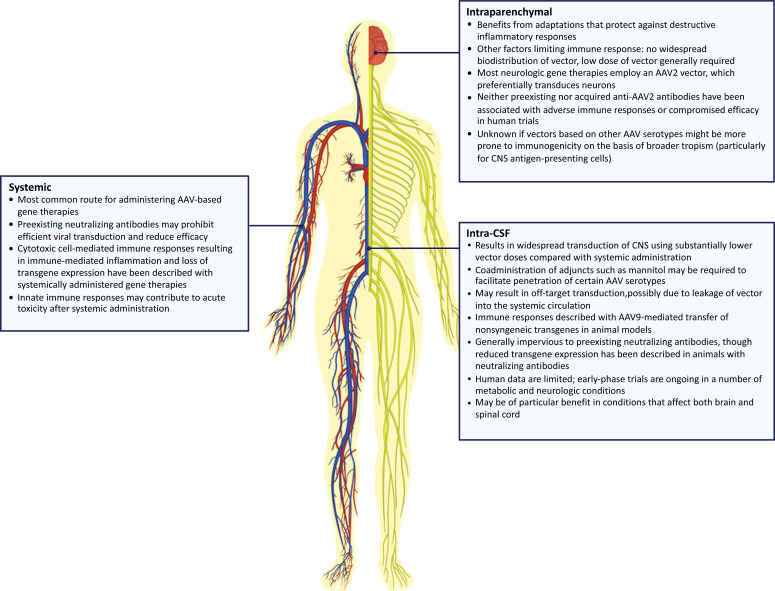
**Features of different sites for gene therapy administration [7,36].** AAV, adeno-associated virus; AAV2, adeno-associated virus serotype 2; AAV9, adeno-associated virus serotype 9; CNS, central nervous system; CSF, cerebrospinal fluid. Colematt *via* Getty Images.

Recombinant adeno-associated viruses (AAVs) are the leading vector platform for *in vivo* gene therapy, with therapies based on AAV serotype 2 (AAV2) and AAV serotype 9 (AAV9) currently approved by the EMA and FDA, partially due to their nonpathogenic nature and low immunogenicity relative to other viral vectors [1-5]. However, ≤80% of the population is seropositive for antibodies against wild-type AAV after the first year of life [6, 7]. Because preexisting immunity may theoretically reduce the efficacy of transduction or trigger an immune response in patients treated with AAV vector–mediated gene therapy [1, 4], screening for neutralizing antibodies and exclusion of seropositive subjects is required in many clinical studies evaluating such therapy [4]. Conversely, administration of recombinant AAV vector may itself elicit an immune response, depending on such factors as an anatomic site of administration (Fig. **1**) [7-36] and vector dosage [7, 28, 36].

AAV vector–mediated gene therapy is often administered systemically for multisystem diseases affecting tissues within or outside the central nervous system (CNS) [30]. Preexisting neutralizing antibodies may interfere with efficient viral vector transduction and efficacy of systemically administered AAV-based gene therapy [17, 23, 24, 26, 28]. Seroconversion of previously seronegative individuals has occurred after systemic administration of AAV vector– mediated gene therapy in clinical trials, potentially precluding future reapplication of the same vector [7, 21, 36]. In addition, dose-dependent cytotoxic immune responses, characterized by expansion of AAV capsid–specific T cells, have been observed in clinical trials of liver-directed, AAV-based gene therapy in hemophilia, resulting in immune-mediated inflammation of transduced cells and loss of transgene expression [7, 24, 26, 28, 29, 36]. Such reactions may be more likely with systemic administration given the need for relatively high vector doses to achieve sufficient transgene expression [30]. Nonspecific innate immune responses may also contribute to acute immune-mediated toxicities after high-dose systemic AAV vector administration, as suggested in preclinical studies and in a patient with Duchenne muscular dystrophy who manifested acute toxicities and complement activation days after receiving systemic AAV-based gene therapy; however, broader clinical evidence delineating the role of innate immune responses in acute immune-mediated toxicities is limited [7, 21, 28, 37-40].

In contrast to the systemic route, localized gene therapy may limit the risk of immunogenicity and potentially eliminate any physiologic barriers to gene transfer (*e.g*., blood –brain barrier) while maximizing vector concentration in proximity to target cells [7, 18, 22, 25]. In the CNS, intrathecal and intracerebroventricular administration of AAV (primarily AAV9) into the cerebrospinal fluid (CSF) in nonhuman primates resulted in widespread viral transduction throughout the brain and spinal cord, at doses ≤30 times less than those used for systemic administration [18, 27, 31, 32]. CSF-administered AAV vector–mediated gene therapy similarly yielded broad transgene expression and therapeutic benefits in animal models of metabolic and neurodegenerative conditions [19, 27, 41, 42]. However, this administration route is associated with off-target tissue transduction [18, 19, 27, 35, 42], potentially resulting from vector leakage into the systemic circulation [18-20]; wider vector biodistribution may increase the risk of immunogenicity [22]. Findings suggestive of possible immunogenicity have been noted in animal models after AAV9-mediated transfer of nonsyngeneic transgenes *via* CSF and corresponded with neurotoxic effects in some cases [16, 33]. Furthermore, CSF administration may not protect against the effects of preexisting peripheral neutralizing antibodies, as evidenced by a nearly complete lack of CNS gene transduction following intrathecal administration of AAV vector–mediated gene therapy in seropositive nonhuman primates [32]. Nevertheless, given the capacity for widespread CNS transduction *via* this route, CSF-administered gene therapy may be particularly advantageous in conditions that affect both the brain and spinal cord [35, 43]. At present, early-phase human trials of such therapy are ongoing for several CNS disorders, including for mucopolysaccharidosis types I [8] and II [9, 10], neuronal ceroid lipofuscinosis [11, 12], GM2 gangliosidosis [13, 14], and giant axonal neuropathy [15]. Other sites such as the subretinal space have been investigated for inherited retinal disorders, with little to no detectable immune response to the therapy [3, 5, 28].

Compartmentalized sites within the CNS and the eye, such as the brain parenchyma and retina, benefit from adaptations protecting them from destructive inflammatory responses [22, 28]. Direct delivery of viral vectors into these sites eliminates or reduces the impact of preexisting humoral immunity, in contrast to systemic delivery [25, 28, 44]. Other factors that may contribute to a lower risk of immunogenicity of vector-based gene therapy administered *via* these sites include avoidance of widespread vector biodistribution and the need for relatively low vector doses (*e.g*., 9.0×10^10^–4.7×10^12^ vg in Parkinson disease (PD) and AADC deficiency) compared with those typically used for systemic (*e.g*., 6.7×10^13^–2.0×10^14^ vg for spinal muscular atrophy), or even CSF-based administration, especially when the targeted area is small and readily isolated [5, 7, 20, 22, 25, 30, 45]. Intraparenchymal delivery of AAV vector–mediated gene therapy has been evaluated in early-phase clinical trials in PD [44, 46-49], Alzheimer disease [3, 50], Canavan disease [51], and aromatic l-amino acid decarboxylase (AADC) deficiency [45, 52], with ongoing trials in lysosomal storage diseases GM2 gangliosidosis [14] and mucopoly saccharidosis type IIIA [22, 53].

## 2 AAV2 VECTOR–MEDIATED GENE THERAPY IN AROMATIC L-AMINO ACID DECARBOXYLASE DEFICIENCY

In intraparenchymal gene therapy, the affinity of certain AAV serotypes to specific cell types has been leveraged to precisely target disease-specific cells while avoiding the transduction of other cells that may elicit an immune response [25]. AAV vector–mediated neurologic gene therapy has been largely unaffected by the immunological response, primarily due to the use of vectors based on AAV2, which preferentially transduces neurons in the CNS [16]. Among clinical trials evaluating direct intraparenchymal gene therapy administration, 67% employ an AAV2 vector [25]. AAV9, another vector of considerable interest in neurologic gene therapy given its ability to transduce astrocytes and neurons, was associated with neurotoxic immune reactions in preclinical studies of intraparenchymal gene therapy, presumably due to off-target transduction of antigen-presenting cells in the CNS [16, 25, 33]. However, clinical experience with intraparenchymal AAV9 vector–mediated gene therapy in humans is limited; therefore, the impact of broader AAV9 cellular tropism in this setting is not well known [25].

**Fig. (2):**
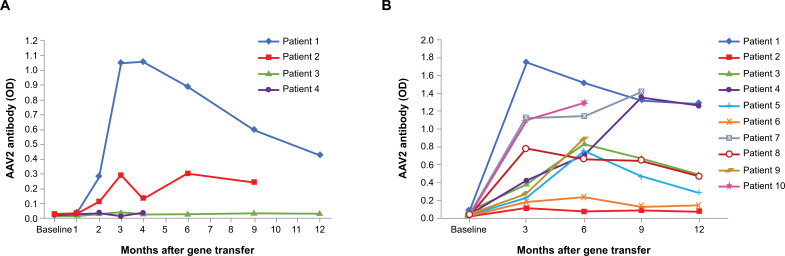
**Antibody titers in patients with AADC deficiency receiving AAV2-hAADC gene therapy.** (**A**) Four patients in 1 trial and (**B**) 10 patients in a later trial received 1.8 × 10^11^ vector genomes, all *via* bilateral intraputaminal infusion. Anti-AAV2 antibody titer was measured at baseline and at specified timepoints ≤12 months after gene therapy. AADC, aromatic l-amino acid decarboxylase; AAV2, adeno-associated virus serotype 2; AAV2-hAADC, AAV2 vector containing the entire coding region of the human *DDC* gene; OD, optical density. Fig. (**2A**) from Hwu, W.L., *et al.*, Gene therapy for aromatic l-amino acid decarboxylase deficiency. *Sci Transl Med*, 2012. 4(134): p. 134ra61. Reprinted with permission from AAAS. (Fig. **2B**) reprinted from *The Lancet Child & Adolescent Health*, 1, Chien YH, *et al.*, 265-173, © 2017, with permission from Elsevier.

AADC deficiency is a rare inherited neurologic disorder resulting from pathological variants in the dopa decarboxylase (*DDC*) gene encoding the AADC enzyme (EC 4.1.1.28). Lack of AADC enzyme leads to a severe combined deficiency of neurotransmitters, including dopamine, serotonin, epinephrine, and norepinephrine [54, 55], resulting in clinical symptoms such as failure to achieve motor milestones, hypotonia, oculogyric crises, delayed speech development, and autonomic dysfunction; these symptoms are apparent in infancy [54, 55]. Gene therapy for AADC deficiency consists of an experimental, intraputaminally administered recombinant AAV2 vector containing the entire coding region of the human *DDC* gene (AAV2-hAADC) [45, 52]. Since most of the AADC activity is found in the striatum [56], local delivery of the *AADC* gene is expected to alleviate symptoms. Indeed, intraputaminal administration of AAV2-hAADC improved motor function in a clinical trial of 4 children with AADC deficiency. In this trial, anti- AAV2 antibody titers were measured at baseline and after treatment with AAV2-hAADC [52]. All patients had a negative antibody titer at baseline, as measured by an enzyme-linked immunosorbent assay (ELISA) method developed for rapid screening of neutralizing antibodies using whole vector particles as antigens. In the ELISA, a neutralizing antibody titer of 1:32 in cell transduction assay corresponded to an optical density (OD) of 0.5 [57]. Antibody titers increased slightly in 2 patients after gene transfer (Fig. **2A**). There was no correlation between antibody titer and clinical outcomes; all 4 patients showed improvements in motor function [52]. A later clinical trial included 10 patients with AADC deficiency. Anti-AAV2 antibody titers were measured at baseline and every 3 months after gene therapy. All patients had a negative antibody titer (≤0.1 OD) at baseline, as measured by ELISA. Antibody titers increased in all patients after gene therapy and declined over time. Antibody titer did not impact motor function improvement (Fig. **2B**) [45]. Results from a study conducted by a separate group of investigators showed improved motor function despite elevated antibody titers (≤1:56–1:28,000) in 6 patients with AADC deficiency at 6 months after treatment with AAV2-hAADC [58]. Although the detailed process of antibody formation against AAV2 capsid remains unknown, leakage of vector particles into the CSF or interstitial fluid during infusion was the most likely mechanism eliciting an immune reaction. Importantly, the antibodies raised against AAV2 capsid did not affect the persistent expression of AADC in the putaminal neurons, as evidenced by increased putaminal uptake of l-6-[^18^F] fluoro-3, 4-dihydroxyphenylalanine, a tracer for the AADC enzyme, on positron emission tomography at 6 [52] and 12 months [45] after AAV2-hAADC treatment.

Clinical trial data in adults with PD lend additional support to the concept that intraputaminal AAV2 vector –mediated gene therapy has a low risk of provoking an immune response. In 3 trials of adult patients with PD (N=29) who received AAV2 vector–mediated gene therapy, eleva- ted anti-AAV2 antibody titers were observed in 10 patients at 6 months, with no impact on therapeutic efficacy or safety [46, 47, 49]. Even in patients with high pretreatment neutralizing antibody titers, therapeutic efficacy was preserved, and there were no safety issues [44]. As previously noted, the prevalence of anti-AAV antibodies is lower in children than adults [6]; therefore, preexisting immunity is less likely to be a factor when AAV2 vector–mediated gene therapy is administered to children, as is the case in AADC deficiency.

## 3 PATIENT MONITORING

Our clinical experience and data we have obtained suggest that anti-AAV2 antibodies are not associated with adverse immune responses and do not compromise the efficacy of AAV2 vector–mediated gene therapy administered to the brain (specifically the putamen); this is likely due to the transduction of target cells prior to the development of an immune response. No patients in the aforementioned clinical trials required pretreatment prophylaxis or posttreatment mitigation of an immune response. Based on the described data, we can conclude that the presence of such pretreatment antibodies should not impact the decision to treat unless symptomatic presentation suggests an elevated risk for an immune response.

## CONCLUSION

The efficacy and safety of AAV vector–mediated gene therapy may be adversely affected by preexisting wild-type anti-AAV antibodies or, rarely, immunogenic reactions to the viral vector. Strategies such as localized administration of low-dose AAV vector–mediated gene to specific sites and, in CNS diseases, the use of vectors based on AAV serotypes that preferentially transduce neurons in the CNS, may reduce the likelihood of adverse immune reactions. Clinical trials of intraputaminally administered gene therapy employing an AAV2 vector in AADC deficiency and PD suggest that such strategies indeed reduce the risk of immune reactions that adversely affect safety or efficacy. Based on these data and our collective clinical experience, we contend that clinical outcomes of AAV2 vector–mediated gene therapy to the brain are not impacted by preexisting or acquired anti-AAV2 antibodies.

## LIST OF ABBREVIATIONS

AADC: Aromatic l-Amino Acid Decarboxylase
AAV: Recombinant Adeno-Associated Viru-ses
AAV2-hAADC: Recombinant AAV2 Vector Containing the Entire Coding Region of the Human *DDC* Gene
AAV9: AAV Type 9
CNS: Central Nervous System
CSF: Cerebrospinal Fluid
DDC: Dopa Decarboxylase
ELISA: Enzyme-Linked Immunosorbent Assay
OD: Optical Density
PD: Parkinson Disease
